# The role of glycosaminoglycan modification in Hedgehog regulated tissue morphogenesis

**DOI:** 10.1042/BST20220719

**Published:** 2023-05-24

**Authors:** Fabian Gude, Jurij Froese, Georg Steffes, Kay Grobe

**Affiliations:** 1 Institute of Physiological Chemistry and Pathobiochemistry, University of Münster, Münster, Germany; 2 Institute of Neuro- and Behavioral Biology, University of Münster, Münster, Germany

**Keywords:** cytoneme, development, Drosophila, glypican, Hedgehog, heparan sulphate

## Abstract

Patterns of gene expression, cell growth and cell-type specification during development are often regulated by morphogens. Morphogens are signalling molecules produced by groups of source cells located tens to hundreds of micrometers distant from the responding tissue and are thought to regulate the fate of receiving cells in a direct, concentration-dependent manner. The mechanisms that underlie scalable yet robust morphogen spread to form the activity gradient, however, are not well understood and are currently intensely debated. Here, based on two recent publications, we review two *in vivo* derived concepts of regulated gradient formation of the morphogen Hedgehog (Hh). In the first concept, Hh disperses on the apical side of developing epithelial surfaces using the same mechanistic adaptations of molecular transport that DNA-binding proteins in the nucleus use. In the second concept, Hh is actively conveyed to target cells via long filopodial extensions, called cytonemes. Both concepts require the expression of a family of sugar-modified proteins in the gradient field called heparan sulphate proteoglycans as a prerequisite for Hh dispersal, yet propose different — direct *versus* indirect — roles of these essential extracellular modulators.

## Introduction

The proteins of the Hedgehog (Hh) family are powerful morphogens that control growth and patterning in different developing tissues in metazoans. In vertebrate embryogenesis, Sonic Hedgehog (Shh) — one of the three members of the Hh protein family (Shh, Indian Hh (Ihh), and Desert Hh) — acts at significant distances from its source during development [1–4], maintains the stem cell niche, including the cancer stem cell niche [5], and progresses various cancers in the adult [5–7]. In *Drosophila melanogaster,* the *hh* segment polarity gene establishes the basis of the fly body plan [8]: Hh is expressed in the posterior (P) compartment of embryonic segments and later in development in the P-compartment of imaginal wing discs before spreading anteriorly to signal to a narrow stripe of tissue neighbouring the anterior/posterior (A/P) compartment border. This results in concentration-dependent expression of target genes in a stripe anterior to the border in the Hh-receiving compartment, including the Hh receptor *patched* (*ptc*) and *engrailed* (*en*, both target genes are expressed close to the Hh source in response to high Hh dose), and *decapentaplegic* (*dpp*, a target gene that responds to low Hh dose) [9,10]. Of note, Hh spreading through the anterior compartment strictly requires unimpaired expression of heparan sulphate proteoglycans (HSPGs) of the Glypican (Glp) family [11–13], and models aiming to explain Hh transport and signalling at distant target sites need to integrate and be able to mechanistically explain these essential Glp HSPG functions.

**Figure 1.**
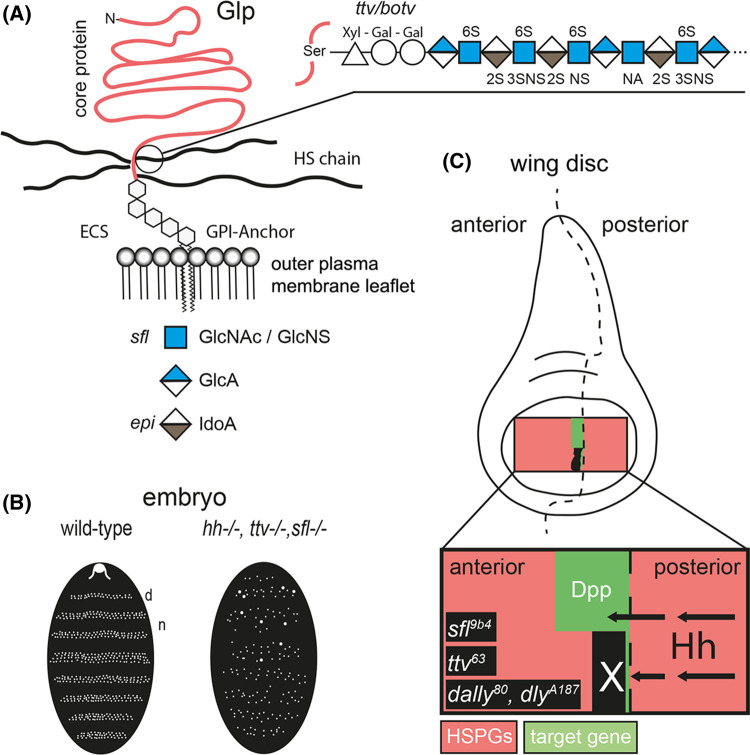
Glp HSPGs are essential for Hh spread. (**A**) HS-decorated Glps localize to the outer plasma membrane leaflet and are exposed to extracellular binders. After biosynthesis of the xylose–galactose–galactose–glucuronic acid linker to a serine side chain of the Glp core protein, HS grows by Ttv/Botv-catalyzed copolymerization of GlcAβ1,4 and GlcNAcα1,4 and undergoes modification by Sfl-mediated removal of acetyl groups from subsets of GlcNAc residues and addition of sulphate to the free amino groups. Subsequent activities of O-sulfotransferases and a GlcA C5-epimerase (Epi) complete HS biosynthesis. (**B**) Scheme of similar patterning defects in *Drosophila* embryos upon inactivation of *hh* or HS biosynthetic enzymes. In both cases, the organization of repeating denticle (d) and naked (n) parasegments in mutant embryos which normally decorate *Drosophila* embryos and first instar larvae is perturbed. (**C**) Schematic of clonal analysis showing that the absence of Glp HSPGs blocks transport of Hh produced in the posterior compartment of the *Drosophila* wing disc to the receiving anterior compartment (arrows) [20]. Instead, Hh accumulates in front of cell clones deficient for the gene sulphateless (*sfl*^9b4^; this mutant expresses undersulphated HSPGs) and *ttv*^63^ (this mutant expresses Glp core proteins without HS chains) in the anterior compartment (marked in black). Of note, Hh fails to cross more than one or two HSPG mutant cells in the anterior compartment (black field with white X), as indicated by the complete absence of *dpp* (green) target gene expression in the clone as well as in wild-type cells anterior to small clones. HS is therefore required for the spreading of Hh in the anterior (e.g. Hh receiving) compartment. Schemes represent findings as described in [16,20].

Glp HSPGs are anchored to the outer leaflet of the plasma membrane via a glycosyl-phosphatidyl-inositol (GPI) anchor and are modified by multiple unbranched heparan sulphate (HS) glycosaminoglycan chains [14] (Figure 1A). The length of extracellular HS chains is ∼100 nm (an average number, lengths vary between 40 nm and 160 nm [15]). In *Drosophila*, HS biosynthesis is initiated by the glycosyltransferase Brother of tout-velu (Botv) and chains are extended with alternating N-acetylglucosamine (GlcNAc) and Glucuronic acid (GlcA) residues by the glycosyltransferase Tout-velu (Ttv) (Figure 1A). Thus, the loss of Botv/Ttv function in the fly results in a complete loss of HS [16,17]. Additional enzymes modify the HS carbohydrate backbone. First, the fly N-deacetylase/N-sulfotransferase, called Sulphateless (Sfl) [18], removes acetyl groups from GlcNAc residues, which are then converted to N-sulphated glucosamine (GlcNS). The N-deacetylated/N-sulphated regions of the HS chain are further modified by a GlcA C5 epimerase that converts GlcA into iduronic acid (IdoA) and 2-O, 3-O, and 6-O sulfotransferases that generate highly negatively charged HS chains (called heparin if produced in connective tissue type mast cells). Extracellular HS sulfation and charge in the fly can also be reduced by the secreted 6-O-endosulfatase DSulf (Sulf1 and Sulf2 in vertebrates [19]). Together, these activities result in HS that binds multiple secreted factors depending on charge or sulfation pattern to regulate their tissue distribution, stability and signalling activities [14].

**Figure 2.**
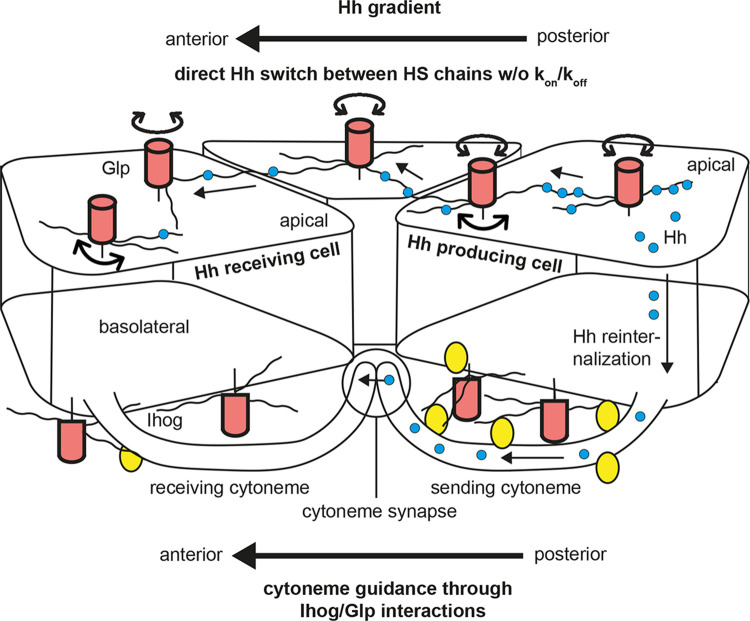
Two mechanistic modes — one indirect, one direct — of HSPG-dependent Hh transport. In polarized producing cells (right), Hh and GPI-anchored Glp HSPGs are first expressed at the apical membrane and assemble in cholesterol- and glycolipid-enriched membrane microdomains (lipid rafts) [28–30]. The first Hh transport mode starts with the HSPG-dependent internalization and transcytosis of apical Hh/Glp clusters to the basolateral side and subsequent cytoneme-mediated anterior transport (bottom) [31–33]. Hh-bearing cytonemes contact target cells directly or, to extend their signalling range, grow towards signal-receiving cytonemes that originate from posterior target cells. Receiving cytonemes express the Hh receptor Ptc and signalling co-factors at specific contact sites (dubbed ‘cytoneme synapses'). The alternative mode posits that Hh associated with Glp HSPGs (Dally/Dlp in *Drosophila*) — that can rotate and freely move on the apical cell surface — transport via Glp-HS mediated direct hand-over between the sugar chains (top) across several cell diameters. Glps are depicted as red cylinders with attached HS chains (black lines), Ihog as yellow circles and Hh as blue circles.

The first indication that HSPGs affect Hh distribution and signalling in *Drosophila* came from genetic screens for segment polarity cuticle phenotypes in embryos (Figure 1B) or loss of *en/ptc/dpp* target gene expression in wing disc tissues (Figure 1C). These screens revealed that Hh function critically depends on Ttv, the fly HS co-polymerase [11,12]. Consistent with this, mutations in the other two HS biosynthetic enzymes, Botv and Sotv, also affect Hh signalling [16,21,22]. HSPG-dependent Hh function was further demonstrated by mutations in Division abnormally delayed (Dally, [23]) and Dally-like protein (Dlp), the two *Drosophila* Glp core proteins that are Ttv substrates. Embryos lacking Dlp activity exhibit defects in Hh distribution and signalling, and both Dally and Dlp regulate Hh movement in the anterior wing disc compartment by a mechanism dependent on their HS chains [20,24]. This dependency was most convincingly supported by mosaic analyses in the *Drosophila* wing disc, revealing that Hh produced in the posterior compartment was unable to cross cell clones in the anterior compartment made deficient in *sfl*, *ttv* or *dally/dlp* gene function (Figure 1C) [20]. Instead, Hh accumulated in front of these cell clones. Notably, Hh was unable to cross HSPG mutant cells, as indicated by the complete absence of *dpp* target gene expression in wild-type cells anterior to Sfl, Ttv or Dally/Dlp deficient wing disc clones. Similar observations were made in vertebrates: When the secreted 6-O-endosulfatase Sulf1 was overexpressed in cells adjacent to a source of green fluorescent protein-tagged Shh (Shh-GFP) in a 32-cell stage *Xenopus laevis* embryo, Shh-GFP transport within the Sulf1 overexpressing region was completely abolished [25]. The lack of observable Shh-GFP in regions where cells express high levels of Sulf1 therefore suggests that Shh is unable to migrate through a vertebrate matrix in which 6-O sulfation of HSPGs is diminished and overall negative HS charge is reduced. Together, these observations strongly support that HS is required for the spreading of Hh/Shh and that HS shapes the morphogen concentration gradient [22,26]. However, since the time these observations were made more than two decades ago, the underlying mechanistic modes of HSPG-dependent Hh/Shh morphogen spread remained a mystery. This is now about to change: Two recently published concepts reveal that direct apical HSPG/Hh interactions and indirect basolateral Glp HSPG interactions with cytonemes — which are actin-rich, filopodia-like cellular extensions that connect Hh-producing and Hh-receiving cells [27] — steer long-range Hh signalling in *Drosophila* wing discs (Figure 2).

## Diffusive Hh transport and the questions it raises

Upon their production in posterior source cells, it has been noted that the simplest mechanism for Hh to spread towards its anterior targets is passive diffusion through the extracellular space, similar to the established passive diffusion of Bicoid and Nanos in early *Drosophila* syncytium embryos [34]. In the extracellular matrix, however, passive diffusion is not sufficient to form functional gradients for two main reasons. The first reason is that movement of molecules that disperse by passive diffusion is fast and efficient over small distances in closed compartments (such as the early *Drosophila* syncytium) but is too slow to reach faraway targets. This is because the timescale of diffusion increases with the square of the distance (*t* = L^2^/D, where *t* is time, D is the diffusion coefficient and L is the distance covered [35–37]). The second reason, which is directly related to the first, is that patterning of folded epithelia like the wing or leg discs in *Drosophila* (that are open compartments because they border fluid-filled spaces) is impossible if the morphogen diffuses away from the plane of epithelial cell layers, as this would prevent most Hh to find its receptor Ptc at distant sites on the same epithelium [38]. To solve these two efficiency problems, it has been suggested that Hh diffusion is restricted to two dimensions on the epithelial plane by reversible interactions with weak, non-signalling interactors, such as the cell-surface HSPGs [39]. Such a surface-restricted transfer mode in which Hh would undergo repeated cycles of HSPG binding, passive diffusion, and rebinding was dubbed ‘hindered diffusion'. Indeed, disrupted HSPG interactions of fibroblast growth factor 2 (Fgf2) were shown to dramatically increase Fgf2 diffusion, demonstrating that HSPGs act as negative regulators of diffusion and Fgf2 spread [40]. Fgf8 [41] or bone morphogenetic protein (BMP) 4 [42,43] also behave in such a manner. However, Hh is clearly not transferred by hindered diffusion, because unimpaired HSPG-interactions *facilitate* Hh spread in the anterior compartment instead of slowing it down [20]. As a consequence, HSPG mutant cells block Hh spread [20], in contrast with promoting the spread of Fgf2, Fgf8 and BMP4. Please refer to [44] for an excellent recent discussion on (HSPG-modulated) diffusion as the transport mechanism underlying gradient formation. However, the paradox of HSPG-*facilitated* Hh spread may also be resolved by non-diffusion based transport modes, for Hh as well as for other morphogens, that are currently intensely investigated. The most prominent of these non-diffusion based transport models is active Hh morphogen relay by cytonemes.

## Ligand-independent, basolateral glypican-Ihog interactions steer Hh-bearing cytonemes

Signalling cytonemes are long (reaching up to 300 µm from the cell body), thin (typically smaller than 200 nm in diameter) and highly dynamic filopodia that facilitate transport of several pattern-generating molecules, such as the BMPs (called Dpp in the fly), Wnt/Wingless, Fgf and Hh across developing tissues [45,46]. Cytonemes that extend from producing cells as well as from receiving cells were first detected in *Drosophila* wing imaginal discs [47]. Later, cytonemes were also found to pattern the zebrafish neural plate [48], to transport Hh in mouse cells [49] and chick limb buds [50], and to transport Wnt to regulate gastric cancer cell proliferation [51]. This indicates that cytoneme-mediated signalling is also central to tissue homeostasis, tumour growth and malignancy. During Hh transport from the posterior compartment of the wing disc epithelium to anterior compartment cells, cytonemes from both compartments make contact on the basal side of the polarized epithelium (Figure 2). These extracellular contact sites are reminiscent of synaptic processes between neurons, and were therefore named ‘cytoneme synapses'. Yet, because Hh ligands initially secrete to the apical membrane of polarized cells and subsequently multimerize using glypican HSPG as scaffolds [30], Hh is not readily available for cytoneme-mediated, basolateral signalling at the synapse. Most evidence so far therefore posits that Hh re-internalizes as a prerequisite for further transport along or within cytonemes [50,52]. Subsequent transport is thought to occur in vesicular form, most likely on multivesicular bodies or exosomes that derive from multivesicular bodies, in an active, actin-motor driven manner [49]. Of note, Ptc receptors are also initially expressed on the apical plane of polarized cells, and therefore also need to internalize and transfer to basolateral sites. There, morphogen-receiving anterior cells or cytonemes present Ptc to Hh sending cytonemes at the synapse [53,54]. The cytoneme transport mode therefore posits both, posterior internalization of Hh ligand and anterior Ptc receptor internalization and association with multivesicular bodies during transport to the basal side. This association is mediated by the endosomal sorting complexes required for transport (ESCRT) machinery, followed by SNAP-receptor (SNARE) mediated vesicle fusion to present Ptc at the membrane of receiving cytonemes. Hh signalling strength and the slope of the gradient in the cytoneme relay system are therefore determined by a complex system of regulated ligand and receptor internalization, transport and externalization. In addition, Hh slope and signalling strength are determined by cytoneme length, the number of contact sites between sending and receiving cytonemes, and by the frequency of contacts made by these highly dynamic structures [55]. This, in turn, leads to the important question of how cytoneme number, stability and length are regulated to specifically steer downstream Hh signalling, and what possible roles HSPGs play in these processes.

The first hypothesis to answer these questions came from the observation that in addition to Hh and Ptc complexes that form at cytoneme synapses, the adhesion molecules and Hh co-receptors Interference hedgehog (Ihog) and Brother of Ihog (Boi) are also expressed on cytonemes and co-localize with the signalling complex [33,55,56]. Vertebrate Ihog/Boi homologues are Cell Adhesion Molecule-Related/Down-Regulated by Oncogenes (CDON) and Brother of CDON (BOC) that are also present on mouse cell cytonemes [49]. These proteins are type I transmembrane proteins with four immunoglobulin-like and two Fibronectin III (FNIII) domains in invertebrates, while vertebrate CDON and BOC consist of five and four Ig-like domains, respectively, followed by three FNIII repeats. One established role of Ihog and Boi in the wing disc is to bind Hh via the first FNIII domain [57,58], and both molecules share redundant functions at the level of the Hh receptor Ptc [59]. In addition, Ihog is known to engage in trans-homophilic binding [60] to stabilize cytonemes, but only until Hh presence disrupts homophilic Ihog/Ihog interactions to enable Hh transport or internalization of the Hh–Ihog–Ptc receptor complex [61]. This mechanism may therefore help determine the number of contact sites between sending and receiving cytonemes and the frequency of contacts.

Importantly, the first Ihog FNIII domain also binds HS/heparin [57], and the ubiquitously expressed Glps Dally and Dlp were also found to be present on Hh-producing cells and on cytonemes. The first established role of Dally interactions with the close Ihog relative Boi was to help recycle Hh from the apical cellular site where it is initially expressed to the basolateral plasma membrane and to basal cytonemes [33]. At the basolateral part of the wing disc epithelium, the second role of HSPGs is to interact with Ihog, Boi and Shifted to factlitate Hh release and its extracellular movement [33]. A third Glp role in Hh signalling was based on the detection of specific Glp interactions with Ihog, and the finding that these interactions stabilize cytonemes in the Hh-receiving field [27]. It was also found that, upon cytoneme contact with the surface of receiving cells, Glps maintained the levels of Ihog, but not of Boi, and that the overexpression of Ihog, but not of Boi, regulates cytoneme dynamics through the interactions with Glps [31]. The regulation of cytoneme dynamics requires Ihog binding to the HS-chains of Glps via the first FNIII domain, but not that of Boi [31]. Therefore, the first FNIII domain plays a novel regulatory role in Ihog/Glp regulated cytoneme stabilization in addition to their more established role in interacting with the Hh ligand [58] or in forming Ihog–Ihog homophilic interaction for cell–cell adhesion [60]. Of note, heparin is required for not only for the heterophilic HSPG–Ihog interaction as described above, but also supports homophilic Ihog/Ihog *trans* interactions *in vitro* [60]. This raises the possibility that both Ihog/HSPG-dependent adhesion modes stabilize cytonemes *in vivo*.

**Figure 3.**
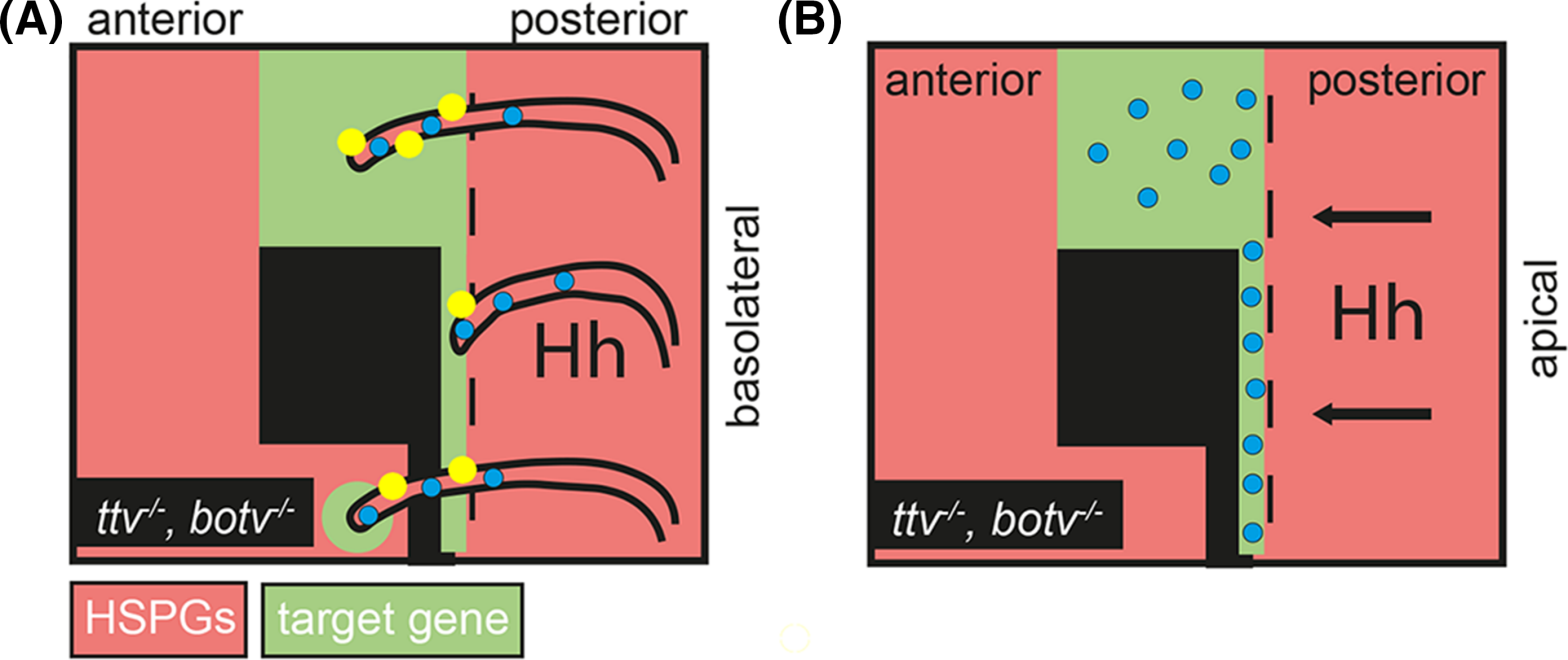
Two Hh transport models to explain the Hhs inability to cross a field of HSPG-deficient cells (labelled black) in the wing disc. (**A**) The cytoneme model posits that Ihog (yellow) expressed on Hh-transporting basolateral cytonemes interact with HSPGs (Hh is shown as blue dots). This interaction is required to stabilize cytonemes. Thereby, lack of HSPGs prevents cytonemes to cross the HSPG-deficient field of cells. Small clones of HSPG mutant cells are crossed, however, and signalling anterior to the clone can be observed (bottom left, [27]). The ability of cytonemes to cross small clones of HSPG-deficient cells, in contrast with endogenously expressed Hh that does not [20], may be explained by Ihog overexpression required for cytoneme visualization in this experimental setup [27,32]. (**B**) The ‘Hh relay' model suggests strongly impaired apical Hh hand-over to clonal cells that do not express acceptor HSPGs or express undersulphated HSPGs. As a consequence, Hh accumulates at the clone border and fails to signal to normal tissue anterior to the clone.

HSPG-regulated Ihog stabilization of cytonemes is especially important because it provided the first explanation as to why Hh cannot properly spread across — and signal behind — anterior HSPG mutant wing disc clones, as described before. Confirmation of the new concept came from the observation that cytonemes labelled with yellow fluorescent protein-labelled Ihog are strongly impaired in their ability to cross *ttv*^−/−^/*botv*^−/−^ compound mutant clones if these clones were located anterior to the A/P compartment border (Figure 3). Likewise, the spreading capacity of less dynamic signal-receiving cytonemes that emanate from the A-compartment of the *Drosophila* wing disc also depended on Glps that were expressed on the plasma membranes of neighbouring cells [55]. As a consequence, the extension of Ihog-expressing cytonemes was significantly reduced when Glp levels in the opposite P-compartment were lowered by knock-down of Dally or Dlp, and cytonemes only rarely crossed through *dally*^−*/*−^/*dlp*^−*/*−^ double mutant clones [55]. Most recently, a mathematical model was developed in which different levels of Ihog and Glps in Hh-producing and Hh-receiving cells dynamically orient and guide cytonemes, and the model was confirmed experimentally in the wing disc [32]. Therefore, taken together, the cytoneme model posits that the spatial information required for cytoneme guidance during Hh reception is provided by Glp/Ihog interactions *in trans* at the cellular interfaces, and that the morphogen is then relayed over short distances to its receptor Ptc at the synapse. This efficiently eliminates the problems associated with diffusive Hh transport over long distances.

## Ligand-dependent, direct HS interactions promote apical Hh transport

As described before, the apparent paradox that HSPG-interactions *facilitate* extracellular Hh transport instead of slowing it down — as is the case for Fgfs or BMP4 — does not support passive or hindered Hh diffusion as a physiologically relevant Hh transfer mechanism. However, two publications recently proposed an HSPG-dependent apical Hh transport mode that is independent of cytonemes and can also solve most of the problems associated with (hindered) diffusion [62,63]. The main hypothesis in these studies is that tradeoff problems of hindered diffusion, as described before, apply only to proteins with one binding site for the HSPG cell-surface interactor. Such proteins need to undergo repeated cycles of HSPG binding and unbinding to intermittently move by passive diffusion at the epithelial plane. Therefore, the tradeoff problem of such a transport mode is that the protein must choose between two possible states: the HSPG bound state that restricts the protein to the epithelial surface (and slows down transport), or the passive diffusion state after HSPG unbinding that allows for protein spread (but increases protein loss from the epithelial plane) [44,62]. The authors hypothesized that a possible solution to this tradeoff problem is to eliminate off-binding and free diffusion steps from the system to permanently restrict the morphogen to an HSPG-mediated continuous transfer mode. This could be achieved by two independent HS-binding sites on the same Hh protein for simultaneous binding of two HSPGs and for direct protein switching between them, which eliminates the ‘off' mode. This, in turn, would allow for uninterrupted, surface-restricted Hh transport. This hypothesis was inspired by the established transport mode of intracellular DNA polymerases, transcription factors, and nucleases that find their target sites by non-specific electrostatic interactions between positively charged amino acids on the DNA-binding protein and the negatively charged DNA backbone [64,65]. The electrostatic attraction allows the protein to move along the axis of the double helix while preventing protein diffusion away from the DNA (a process called ‘sliding’). In addition, these proteins directly transfer from one DNA backbone to the next in a process called ‘intersegmental protein transfer' [64]. Intersegmental protein transfer requires two independent DNA-binding sites to eliminate the need to unbind the first DNA before the second DNA can be bound, thus allowing for continuous protein movement along and between adjacent DNAs. This, in turn, increases the distance L covered by a given number of steps d, and thus the distance the molecule can travel within a given time [66–69].

Both recent studies [62,63] provide evidence that electrostatic interactions between negatively charged HS and two basic HS-binding sites guide extracellular Hh transport in a similar way to maximize Hh search efficiency for its target Ptc (Figure 2). The experimental strategy used in these studies consisted of site-directed mutagenesis of one of two established HS-binding sites of Hh [70,71] and Shh [70,72] into neutral alanines or acidic glutamates to impair the proteins’ capacity for intersegmental protein transfer and to convert Hh into a classic on/off binder with only one fully functional HS-binding site. In the first study [62], Hh and both Hh variants were expressed under endogenous Hh promotor control in *Drosophila* eye- and wing discs made null for endogenous Hh function [73] and analyzed for their ability to form functional gradients *in vivo*. In this system, the expression of wild-type *hh* cDNA under endogenous control fully restored the Hh null fly phenotype. In stark contrast, expression of both HS-binding deficient Hh variants under the same endogenous promotor control resulted in the selective loss of eye and wing tissue known to require HSPGs for long-range Hh spread. In contrast, other fly tissue from the same discs that do not require HSPGs for Hh short-range signalling developed normally. These findings demonstrated that the activity of all Hh variants at the level of Ptc was not affected and confirmed the importance of unimpaired direct Hh/HSPG interactions for Hh spread over longer distances *in vivo*.

To mechanistically explain the *in vivo* findings, Quartz crystal microbalance with dissipation monitoring (QCM-D) was used. The QCM technology is based on an oscillating quartz crystal sensor with a resonance frequency related to its mass. This allows for the real-time detection of nanoscale mass changes on the sensor surface in real-time by monitoring changes of the resonance frequency. The QCM technology can therefore serve as a proxy for Hh interactions with cell-surface HS upon linkage of heparin — representing the most highly sulphated form of HS — to fluid supported lipid bilayers at the sensor surface. Like HS attached to GPI-linked Glps on the cell-surface, heparin coupled to supported lipid bilayers can rotate freely and move laterally on the sensor. The addition of purified unlabelled Shh to the heparin-functionalized surface decreased the resonance frequency upon binding, as expected, and most proteins remained bound during extended washing with buffer. Importantly, the QCM-D technology measures an additional parameter, the change in energy dissipation *D*, which indicates changes in the supported lipid/heparin layer stiffness. This additional parameter revealed that Shh effectively cross-links HS/heparin chains, consistent with the presence of two functional Shh binding sites [72]. In contrast, the ability of Shh mutants with the basic HS-binding lysine [72] exchanged for a neutral alanine or an acidic glutamate to cross-link heparin, e.g. to bind two sugar chains at once, was strongly reduced. Again, this finding showed that both functional Shh binding sites bind two HS/heparin chains simultaneously [72], either permanently (which would fix the protein at the surface) or transiently as an intermediate during Shh movement from one sugar chain to the next — a property that could explain their impaired *in vivo* spread [20]. To distinguish between these two possibilities, soluble heparin was added to the wash buffer to serve as a potential acceptor, and it was observed that soluble heparin rapidly eluted most Shh from the QCM-D sensor surface — but not the mutants. Of note, equal amounts of selectively de-N-sulphated soluble heparins (that lacks sulphates requiring *sfl* activity during biosynthesis), de-6O-sulphated soluble heparin, and de-2O-sulphated soluble heparin did not elute Shh from the sensor surface. These experiments showed that Shh moves quickly and directly between sugar-sulphate chains of equal overall charge, but does not switch from higher sulphated chains to lower sulphated chains or in the absence of acceptor HS — as indicated by stable Hh sensor surface interactions during extended washing in the absence of soluble heparin. These findings can therefore alternatively explain why Hh cannot spread through clones deficient in the HSPG biosynthetic genes *sfl*, *ttv*, and *dally/dally-like* in the *Drosophila* wing disc and why Hh accumulates at, and only signals to cells directly at the clone border (Figure 3) [20].

In the second study [63], the authors used the Gal4/UAS overexpression system to show that Hh variants with one HS-binding site exchanged for neutral alanines caused highly variable, and often striking, wing mispatterning phenotypes caused by altered gradient range and ectopic signalling *in vivo*. These findings are consistent with Hh conversion into a classic on/off binder with only one fully functional HS-binding site that unbinds more easily from HSPGs and undergoes passive diffusion in the bordering fluid-filled space. This, in turn, induces ectopic signalling in tissues that also border this space but normally do not receive Hh. Consistent with these *in vivo* findings, QCM-D confirmed extensive loss of the Hh variant from the heparin-functionalized sensor surface. These findings provide a teleological purpose for the initial step of HSPG-mediated apical multimerization of Hh on producing cells [30], as this prevents Hh loss from the epithelial surface [74] and represents the starting point for direct Hh ‘hand-off' from one HS chain to the next. Therefore, in this model, HSPGs can alter the range of morphogen gradients by modulating both, the extent of morphogen spread and the extent of Hh leakage.

In summary, Hh gradient dynamics, robustness, and scalability in different developing tissues at different time points may be achieved by more than one HSPG-dependent relay mode, e.g. in a direct Hh hand-over mode or an indirect mode via Ihog on cytonemes. Both modes may act together in the same tissue, thereby complementing each other, in parallel, or alternatively in different tissues. One factor that might decide between these possibilities are biophysical properties of the matrix: direct Hh relay may be the preferred way to transmit the signal through dense and stiff matrices, while cytonemes may preferably act in softer tissues. Another factor that might decide between both modes may be the ligand: cytonemes may preferably transport dual-lipidated, plasma-membrane associated Hh, while apically expressed HSPGs may preferably transfer solubilized Hh forms [71,75,76], such as Shh released by the soluble glycoprotein Scube2 [77].

## Perspectives

1. In addition to the Hhs, several other molecules form HSPG-dependent gradients: Wingless (Wg, the *Drosophila* Wnt), Decapentaplegic (Dpp, the *Drosophila* BMP), the Fgfs and chemokines to steer inflammatory processes [78]. It is therefore possible that these molecules also spread by HSPG-mediated hand-over and/or cytoneme-mediated transport.
2. In the past decade, it has become increasingly clear that dynamic repeated cycles of cytoneme extension and retraction can conceivably ‘dot’ the Hh morphogen onto receiving cells, or relay Hh through cytoneme synapses that connect sending and receiving cytonemes. It has also become clear that Hh diffusion and HSPG-mediated transfer between HS chains add to the Hh relay repertoire.
3. Important open questions are whether vertebrate Glps contribute to cytoneme stabilization or direct ligand hand-over in a manner similar to that found in the *Drosophila* wing disc, how gradients generated by both modes can be scaled, and whether gradient formation in other physiological contexts — for example chemokine gradient formation during inflammation — relies on the same principles.

## Abbreviations

BOC: Brother of CDON
CDON: cell adhesion molecule-related/down-regulated by oncogenes
FNIII: Fibronectin III
GPI: glycosyl-phosphatidyl-inositol
HS: heparan sulphate
HSPGs: heparan sulphate proteoglycans
QCM-D: Quartz crystal microbalance with dissipation monitoring
Shh-GFP: green fluorescent protein-tagged Shh
